# Paving the Way to Elucidate Hg's Role in Tumorigenesis

**DOI:** 10.1002/advs.202514828

**Published:** 2026-03-09

**Authors:** Shouying Li, Rong Zhong, Zhenyang Yu, Hualing Fu, Jin Chen, Jia Wei, Ningwei Zhao, Le Qu, Tiantian Li, Wenli Tang, Huan Zhong

**Affiliations:** ^1^ School of Environment State Key Laboratory of Water Pollution Control and Green Resource Recycling Nanjing University Nanjing P. R. China; ^2^ Department of Epidemiology and Biostatistics and Ministry of Education Key Lab of Environment and Health School of Public Health Tongji Medical College Huazhong University of Science and Technology Wuhan P. R. China; ^3^ State Key Laboratory of Pollution Control and Resource Reuse Key Laboratory of Yangtze River Water Environment Ministry of Education College of Environmental Science and Engineering Tongji University Shanghai P. R. China; ^4^ Research Center for Eco‐Environmental Sciences China Chinese Academy of Sciences Beijing P. R. China; ^5^ Center For Global Health School of Public Health Nanjing Medical University Nanjing P. R. China; ^6^ Jiangsu Province Engineering Research Center of Antibody Drug Key Laboratory of Antibody Technique of National Health Commission Nanjing Medical University Nanjing P. R. China; ^7^ Department of Oncology Nanjing Drum Tower Hospital Affiliated Hospital of Medical School Nanjing University Nanjing P. R. China; ^8^ China Exposomics Institute Shanghai P. R. China; ^9^ Affiliated Hospital of Nanjing University of Chinese Medicine Nanjing P. R. China; ^10^ Department of Urology Jinling Hospital Affiliated Hospital of Medical School Nanjing University Nanjing P. R. China; ^11^ National Key Laboratory of Intelligent Tracking and Forecasting for Infectious Diseases National Institute of Environmental Health Chinese Center for Disease Control and Prevention Beijing P. R. China; ^12^ China CDC Key Laboratory of Environment and Population Health National Institute of Environmental Health Chinese Center for Disease Control and Prevention Beijing P. R. China; ^13^ China Meteorological Administration Key Laboratory of Meteorological Medicine and Health National Institute of Environmental Health Chinese Center for Disease Control and Prevention Beijing P. R. China

**Keywords:** cancer, exposure, health, mercury, pollution

## Abstract

Tumorigenesis can be induced by diverse environmental carcinogens, with mercury (Hg)—a global pollutant that accumulates in humans throughout life, crosses the blood‐brain and placental barriers, and is poorly excreted—classified as a Group 2B carcinogen; however, its contribution to human tumorigenesis remains insufficiently characterized. This critical knowledge gap stems primarily from confounding effects of human co‐exposure to other carcinogens and detoxifying agents, coupled with unclear correlations along the external exposure‐internal Hg burden‐tumorigenesis continuum due to insufficient organ‐specific Hg data in humans. To improve the understanding of Hg's role in tumorigenesis, we propose a research paradigm that integrates: 1) advancing Hg speciation analysis in human tissues and internal dynamics of Hg through in vivo transformation studies, to map organ‐specific Hg distributions; and 2) constructing a comprehensive database of demographic factors, Hg exposure profiles, and tumor biomarkers for artificial intelligence‐driven analyses to disentangle Hg‐specific effects from confounding factors. Bridging the gap between Hg exposure and tumorigenesis could help identify overlooked tumorigenic factors, advance targeted prevention strategies, and guide decision‐making in environmental and public health, ultimately improving public health outcomes.

## Graphical abstract

Tumorigenesis can result from diverse environmental carcinogens. Among them, mercury—a lifelong bioaccumulative Group 2B carcinogen—has tumorigenic potential that remains poorly understood due to confounding co‐exposures and limited organ‐specific data. To bridge this gap, we propose a paradigm combining in vivo Hg speciation mapping with AI‐driven multi‐source data analysis to clarify mercury's role in tumorigenesis and guide public health.

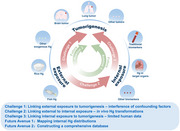

## 1 Introduction

Tumors, which can be benign or malignant, result from uncontrolled cell proliferation, with malignant tumors (i.e., cancer) having the potential to invade tissues and metastasize [1, 2]. Cancer is a leading global cause of death, responsible for 9 700 000 deaths in 2022 [3]. Tumor initiation and progression are strongly influenced by carcinogenic factors, among which environmental pollutants represent an important source of exogenous carcinogens. According to estimates from the World Health Organization (WHO) and International Agency for Research on Cancer (IARC), environmental toxic exposures account for 7%–19% of human cancers [4]. These pollutants in air, water, and soil can enter the human body through inhalation, ingestion, and dermal absorption, thereby increasing tumor risk. For instance, air pollution has been linked to approximately 30% of lung cancer‐related deaths globally [5].

Notably, mercury (Hg) is a ubiquitous environmental pollutant to which nearly all humans are chronically and inevitably exposed via inhalation and dietary intake [6, 7], and it has been classified as a Group 2B carcinogen—possibly carcinogenic to humans—by the IARC since 1993 [8]. This classification was primarily based on carcinogenicity data on Hg from human, experimental animal, and cellular studies [9], with evidence showing that Hg exposure induces oxidative stress and inflammation, abnormal cell proliferation and enhanced survival, and epigenetic alterations—all hallmarks of tumorigenesis (see Section 2 and Table S2 for more details) [10, 11, 12]. Moreover, based on animal studies, Hg is hypothesized to contribute to tumorigenesis in a stepwise manner: Hg exposure may up‐regulate reactive oxygen species (ROS) production through NADPH‐oxidase activation, which can activate nuclear factor erythroid 2‐related factor 2 (Nrf2) signaling and subsequently inhibit apoptosis and autophagy, thereby promoting tumor progression [13]. However, epidemiological studies to date have yielded inconsistent results regarding the association between Hg exposure and tumorigenesis (see Section 2 and Table S2 for more details). While these inconsistencies persist regarding Hg's role in tumorigenesis, recent studies have highlighted Hg's effects on other health issues. For example, even low doses of Hg (25–100 nm—concentrations commonly found in rice and fish [14, 15]) can trigger neuronal degeneration and impair neurological function [16]. In murine embryonic stem cells, exposure to 25 nm methylmercury (MeHg) downregulated transcription‐ and development‐related genes while upregulating genes related to neurodevelopment and cell movement [17]. Exposure to 10–50 nm HgCl_2_ in *Pelteobagrus fulvidraco* decreased lymphoid cells and increased melano‐macrophage centers [18]. Epidemiological studies further indicated that Hg exposure contributed to autism spectrum disorders [19] and showed that prenatal exposure to MeHg at environmentally relevant levels increased the risk of neurotoxicity in infants [20, 21]. These findings, although not specifically focused on Hg's role in tumorigenesis, demonstrate that long‐term exposure to environmentally relevant levels of Hg can pose substantial health risks to humans, potentially including tumorigenesis. This concern is heightened by the lifelong accumulation of Hg in humans and its inefficient elimination. Specifically, when ingested, MeHg typically exhibits an assimilation efficiency exceeding 80% [22] yet minimal daily elimination (∼1.4%) [23], leading to prolonged tissue retention and progressive bioaccumulation. Adding to this concern, future climate scenarios may further exacerbate this issue. Ocean warming has already increased tissue MeHg concentrations in Atlantic bluefin tuna by 56% from 1969 to 2017 [24]. Moreover, modeling studies predict that climate change would raise MeHg levels in freshwater wild fish across Asia by about 60% during 2031–2060 [25]. With over three billion people relying on aquatic products for nutrition, this rise will consequently increase human dietary exposure to Hg. Taken together, the combination of chronic and inevitable human Hg exposure, low excretion capacity for Hg, and climate change‐enhanced dietary Hg bioaccumulation underscores Hg's tumorigenic potential, highlighting the critical need to elucidate its role in population‐level tumorigenesis.

Therefore, elucidating Hg's role in tumorigenesis is essential for informing targeted cancer prevention strategies. To this end, we synthesized existing epidemiological and experimental evidence to delineate Hg's potential role in tumorigenesis, identified critical knowledge gaps, and proposed a systematic research framework for uncovering unrecognized tumorigenic factors.

## 2 Current Understanding of Hg's Role in Tumorigenesis

**FIGURE 1:**
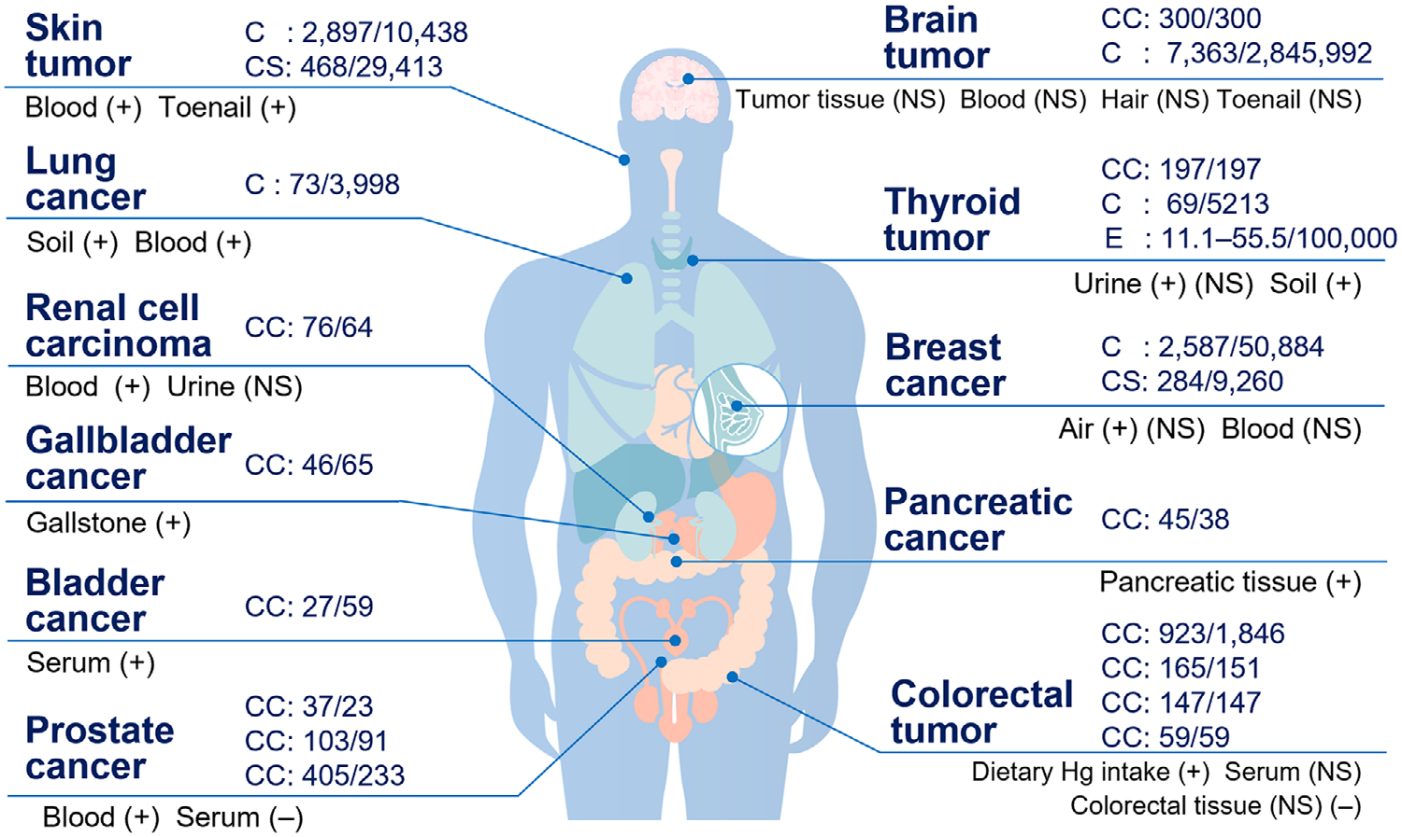
Epidemiological evidence of the potential association between Hg exposure and tumor incidence. “N.A.” indicates that population data are unavailable. The symbols “+”, “NS”, and “–” represent the correlation between tumor incidence and Hg concentrations (in environmental or biological samples) as significantly positive, non‐significant, and significantly negative, respectively. Study types are defined as follows: CC (Case‐Control studies): cases/controls; C (Cohort studies): incident cases/follow‐up population; CS (Cross‐Sectional studies): prevalent cases/surveyed population; E (Ecological studies): cases/reference population. Data sources are listed in Table S1.

The potential role of Hg in human tumorigenesis was proposed as early as the 1980s, when Kazantzis reviewed existing data and found that Hg exposure showed significant genotoxicity, although its carcinogenic effects remained unclear [26]. In recent decades, emerging evidence has shown associations between Hg exposure and tumorigenesis in organs such as the brain, intestines, liver, and kidneys (Figure 1; Table S1), primarily through two lines of evidence: epidemiological studies and experimental models. Both lines of evidence focus on the impact of Hg exposure, distinguishing between external and internal exposure, on tumorigenesis. External exposure refers to inhalation and dietary intake, and is evaluated based on Hg levels in environmental matrices. Internal exposure depends on the Hg burden resulting from Hg absorption, distribution, and transformation among Hg species within the body, and is generally assessed via biomarkers such as Hg levels in blood, hair, and urine. Currently, various epidemiological and experimental studies have examined the relationship between external/internal Hg exposure and tumorigenesis, as detailed below.

Epidemiological studies, including case‐control, cohort, cross‐sectional, and ecological studies (Figure 1; Table S1), are commonly used to investigate associations between Hg exposure and tumor incidence. Among these epidemiological approaches, case‐control designs, which rely on retrospective comparisons, have frequently been applied. For instance, an Indian study of 76 cases and 64 controls reported significantly higher blood Hg concentrations in patients (3.67 ± 3.54 µg/L) with renal cell carcinoma than in matched controls (0.36 ± 0.91 µg/L), implicating Hg's involvement in the development of renal cell carcinoma [27]. However, another case‐control study conducted in Pakistan, focusing on colorectal cancer, measured Hg concentrations in tumor tissues (43 µg/kg, range: 1–715) and adjacent non‐tumor tissues (61 µg/kg, range: 4–972) and found no significant difference, suggesting that Hg accumulation in colorectal tissues may not be directly linked to tumor development in this population [28]. Furthermore, differences in Hg distribution across biological matrices may partly explain the inconsistent associations observed in these studies. For example, some studies have reported a positive correlation between whole blood Hg levels and prostate cancer, whereas Hg levels in serum showed a negative correlation (Figure 1). This discrepancy likely arises because whole blood contains erythrocytes and other cellular components that can bind and sequester more Hg, especially MeHg, while serum represents only the cell‐free, extracellular fraction, resulting in opposite trends between the two matrices. Beyond case‐control designs, prospective associations have also been documented in large‐scale cohort studies. For instance, the Nurses’ Health Study (1984–2012) and the Health Professionals Follow‐up Study (1986–2012) linked elevated toenail Hg levels to increased risks of basal cell carcinoma, squamous cell carcinoma, and melanoma [29]. By contrast, in a historical cohort study of 3 998 Hg miners in Spain (1895–1994), occupational Hg exposure was not associated with increased lung cancer mortality, highlighting that associations may differ depending on cancer type, population, or exposure route [30]. Another example of epidemiological investigation is a cross‐sectional study of NHANES data from 29,413 U.S. participants, which identified significant associations between total Hg (THg)/MeHg levels and non‐melanoma skin cancer prevalence, with odds ratios (OR) of 1.79 [95% confidence interval (CI): 1.19–2.71; *p* = 0.004] for THg and 1.74 (95% CI: 1.13–2.70; *p* = 0.01) for MeHg [31]. In addition, ecological studies examining population‐level data—such as one covering 34.6% of the U.S. population—revealed a positive correlation between regional Hg emissions and estrogen receptor‐positive breast cancer incidence [32]. It is important to note such ecological studies reflect regional trends and may not directly represent individual exposure‐response relationships, as Hg undergoes complex transformation, bioaccumulation, and trophic transfer before entering humans via diet. These epidemiological studies provide initial evidence of the association between Hg exposure and tumor incidence at the population level. However, this relationship may be influenced by regional disparities, co‐exposures modifying Hg toxicity, and potential selection or information biases, as discussed in Section 3.

**FIGURE 2:**
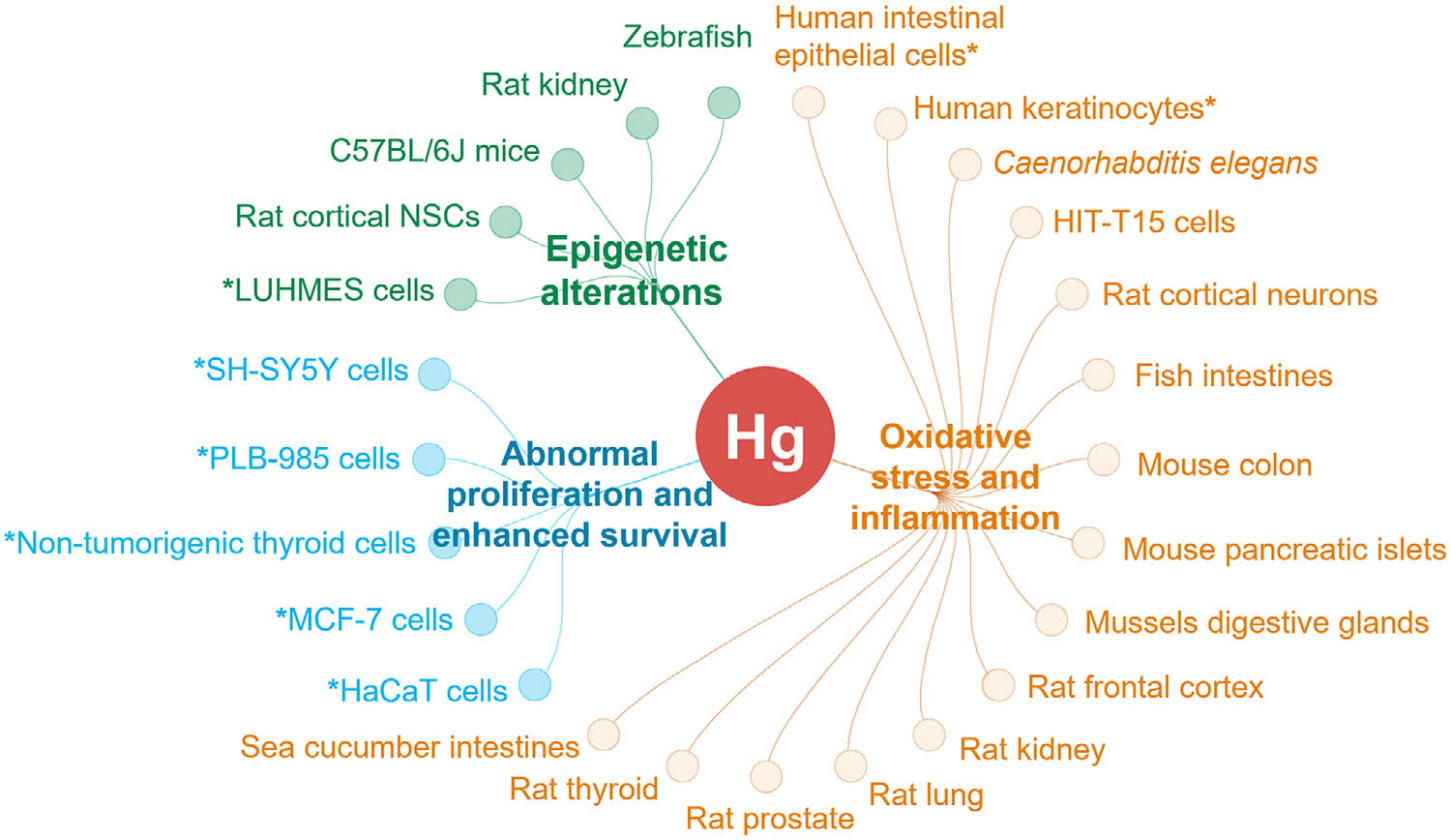
Potential pathways of Hg‐induced tumorigenesis and the corresponding experimental models used to investigate them. Asterisks (*) denote human cells. Data sources are listed in Table S2.

By contrast, experimental models, including animal experiments (in vivo) and cellular models (in vitro), are commonly employed to explore the potential tumorigenic effects of Hg and the underlying mechanisms (Figure 2). Results of these studies suggested that Hg could contribute to tumorigenesis in three ways: 1) inducing oxidative stress and inflammation [10, 16, 33], 2) promoting abnormal cell proliferation and enhanced survival [34], and 3) triggering epigenetic alterations [12] (Figure 2 and Table S2). Oxidative stress caused by the overproduction of ROS led to oxidative DNA damage, which promoted tumorigenesis [35]. For example, exposure to 1 and 5 µm MeHg or inorganic Hg (IHg) for 1 day exacerbated H_2_O_2_‐induced oxidative damage to both mitochondrial and nuclear DNA in *Caenorhabditis elegans*, directly linking oxidative stress to Hg‐induced genotoxicity [36]. In addition, in vivo exposure of Wistar rats to 1.5–15 nm MeHg increased DNA damage in the frontal cortex, providing tissue‐level evidence of Hg‐induced oxidative DNA damage [33]. Similarly, exposure of the human breast cancer cell line MCF‐7 to 1 µm IHg for 1 day promoted tumor cell proliferation through activation of estrogen receptor‐α (ERα) signaling, a key driver of proliferation in estrogen‐responsive breast cancer cells [37]. Furthermore, Go et al. utilized Lund Human Mesencephalic (LUHMES) cells exposed to 1 nm Hg for 6 days—an environmentally relevant concentration—and found that Hg exposure increased DNA methylation [12], potentially contributing to a higher risk of oncogenesis. These experimental models demonstrate Hg's tumorigenic potential through cellular‐ and tissue‐level evidence. In addition to these cellular effects, Hg exposure alters immune‐cell functions: it disrupts T lymphocyte and B lymphocyte signaling [38, 39], impairs macrophage cytokine responses, suppresses neutrophil phagocytosis, reduces natural killer (NK) cell activity, and may shift the Th1/Th2 balance toward Th2 dominance [40]. Such immune dysregulation may weaken tumor surveillance and, together with Hg‐induced oxidative stress and chronic inflammation, contribute to a microenvironment that favors tumorigenesis. Notably, some studies employed high‐concentration, short‐duration exposures that differ from the low‐concentration, chronic exposures typically encountered by humans in real‐world environments. Therefore, the results of these experiments should be interpreted with caution, and future studies should use environmentally relevant Hg concentrations to enhance generalizability. In addition, current mechanistic studies lack systematic investigation of dose‐time/kinetic relationships, which limits our understanding of how exposure intensity, duration, and timing influence mechanistic outcomes and tumor risk. This gap hampers identification of intervention windows and strategies, highlighting the need for further research, which could draw on non‐cancer Hg interventions such as reducing exposure (e.g., avoiding high‐Hg seafood or contaminated areas) [41] and mitigating oxidative stress via selenium supplementation [42] or 2,3‐dimercapto‐propanesulphonate (DMPS) chelation [43]. Moreover, the limited involvement of human subjects in these studies further constrains the direct relevance of the findings to human tumorigenesis, as discussed in Section 3.

## 3 Challenges in Revealing Hg's Role in Tumorigenesis

**FIGURE 3:**
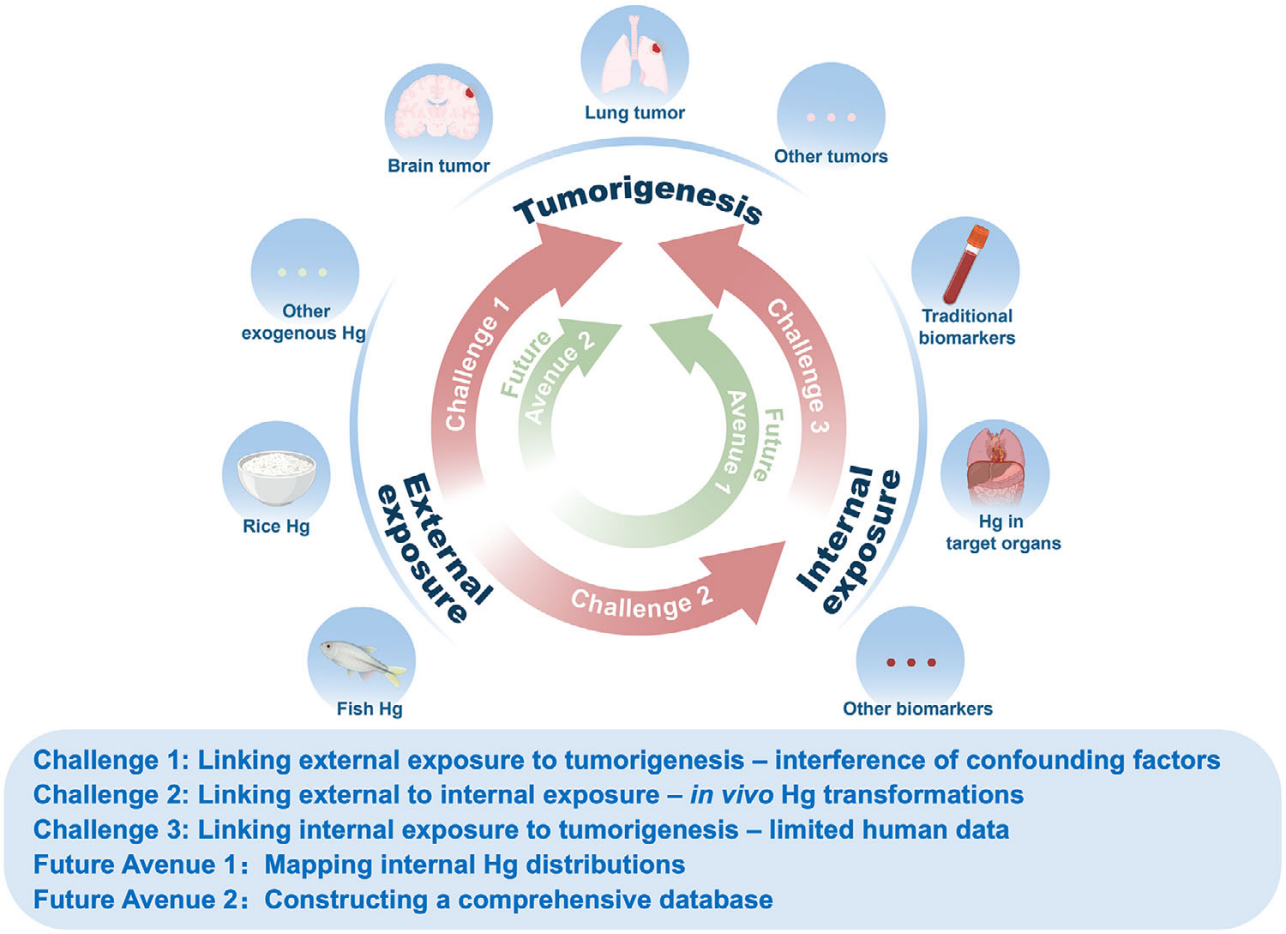
Challenges and future avenues in identifying the role of Hg in tumor risk. Created with BioRender.com.

Despite the aforementioned evidence from epidemiological studies and experimental models, critical barriers remain in determining whether and how Hg induces tumorigenesis. A key challenge in confirming Hg's tumorigenic role stems from confounding factors that mask its specific contribution (see Challenge 1 in Figure 3). Additionally, the extent of Hg accumulation in target organs following external exposure remains unclear, introducing uncertainties in both the external to internal exposure correlation (see Challenge 2 in Figure 3) and the internal exposure to tumorigenesis relationship (see Challenge 3 in Figure 3). Collectively, these unresolved issues hinder elucidating Hg's role in tumorigenesis, as discussed below.

The confounding factors, including other tumorigenic factors and detoxifying agents for Hg, lead to substantial uncertainty in quantifying Hg's contribution to tumorigenesis. On the one hand, humans are routinely exposed to multiple tumorigenic factors (e.g., dust and UV radiation) alongside Hg. Such co‐exposure during critical exposure windows may contribute to tumorigenesis and lead to overestimation of Hg's specific tumorigenic effects. For instance, in mining occupations, workers face concurrent exposure to both Hg and silica dust—a recognized carcinogen associated with elevated lung cancer risk. A cohort study of workers from Hg mines in Spain, Slovenia, Italy, and Ukraine revealed an increased lung cancer risk, which was mainly attributed to co‐exposure to crystalline silica and radon, rather than Hg alone [44]. Similarly, co‐exposure to Hg and arsenic could exacerbate DNA damage through shared oxidative stress pathways [10, 45], and such effects are often statistically attributed to “combined exposure effects” [46]. This makes it difficult to quantify the specific contribution of each factor, thereby significantly increasing the complexity of causal attribution. On the other hand, certain elements and compounds—such as selenium and ascorbic acid—can mitigate the adverse effects of Hg [47]. These substances could enter the human body through dietary intake or inhalation—either concomitantly with Hg or separately—potentially altering Hg's bioavailability, in vivo transformations, and ultimate adverse effects including tumorigenesis. For instance, selenium, often co‐ingested with Hg via fish consumption, mitigated Hg toxicity through the formation of Hg‐selenide complexes in organs such as the liver [48]. Therefore, these confounding factors may obscure the evaluation of Hg's carcinogenic potential in external exposure‐tumorigenesis analyses.

The absence of data on organ‐specific Hg levels presents a significant barrier to accurately assessing internal exposure and its role in tumorigenesis, because organ‐specific Hg directly measures accumulation in target organs and thus serves as the most direct indicator linking Hg exposure to tumorigenesis in these organs. As discussed above, organ‐specific Hg accumulation reflects both external exposure (mainly through ingestion/inhalation) and internal redistribution, thus diverging from traditional biomarkers like Hg levels in blood, hair, urine, or toenails. For example, Hg levels in toenails, though commonly used as a biomarker, have shown poor correlation with Hg levels in internal organs such as the pituitary and thyroid glands [49]. This divergence renders traditional biomarkers inadequate for accurately reflecting Hg levels in target organs and evaluating the tumorigenic potential of Hg exposure. This inadequacy, coupled with the current lack of internal exposure data—such as organ‐specific Hg levels—hampers efforts to link external and internal Hg exposure and to correlate internal exposure with tumorigenesis.

Notably, in vivo Hg transformations may further complicate the role of Hg in tumorigenesis. Generally, THg concentration in biological samples of humans is widely used as a biomarker of Hg exposure, while only a few studies have measured the concentrations of neurotoxic MeHg and other Hg species. This might be attributed to the fact that MeHg is much more easily absorbed after entering the human body; by contrast, IHg is less bioavailable, with 93% being excreted without entering the systemic circulation [39]. Consequently, a number of previous studies have generally assumed that measuring THg—or in some cases MeHg—alone is sufficient to reflect the overall Hg burden in the human body, potentially overlooking the contribution of other Hg species. However, the existing data show that the percentage of MeHg in human blood ranges from 22% to 97% [50, 51], indicating unexpectedly high proportions of IHg. This observation, together with the low bioavailability of IHg from dietary sources, suggests that external exposure alone may not fully explain the presence of IHg. Additional sources, such as in vivo oxidation of elemental Hg [Hg(0)] and demethylation of MeHg, may also contribute. In fact, in vivo Hg transformations in humans have been proposed, including the oxidation of Hg(0) in blood, the methylation of divalent Hg [Hg(II)] in HeLa cells, and the demethylation of MeHg in glioblastoma cells. Although Hg transformations in human organs have not been extensively reported, this phenomenon has been observed in the brain tissues of marmoset monkeys, Sprague‐Dawley rats, and dolphins, suggesting it may also occur in humans [47]. The drivers of these in vivo transformations, such as catalase [52], superoxide anions [53], and selenium, are ubiquitous within the human body [48]. However, although the Hg transformations driven by the aforementioned factors are widely distributed in environments, they have received limited investigation in animals, except in a few organs, such as the brains and livers of rats and monkeys [54, 55, 56], and no relevant studies have been conducted in humans in vivo. As a result, little is known about Hg transformations in organs with high cancer incidence, such as the lungs, prostate, and breast. Furthermore, since THg is the parameter measured in biomarkers and Hg can undergo transformation in vivo, it remains unclear which form of Hg—MeHg or Hg(II)—exerts adverse impacts at the molecular level and thereby contributes to tumorigenesis.

## 4 Future Avenues for Identifying Hg's Role in Tumorigenesis

To help determine Hg's potential role in tumorigenesis, we propose two of the most pressing avenues, based on the challenges outlined above: 1) Mapping internal species‐specific Hg distributions to illuminate the link between external and internal Hg exposure and to assess the association of internal Hg species with tumorigenesis (see Future Avenue 1 in Figure 3), and 2) constructing a comprehensive database that accounts for confounding factors to evaluate the specific role of external Hg exposure in tumorigenesis (see Future Avenue 2 in Figure 3). These efforts collectively establish a targeted strategy to advance epidemiological and mechanistic understanding of the Hg exposure‐tumor risk relationship.

### 4.1 Mapping Internal Hg Distributions

Elucidating the relationship between external and internal Hg exposure, as well as the association between Hg species in human tissues and tumorigenesis, requires a comprehensive understanding of in vivo Hg distributions. Central to this understanding is elucidating Hg's transformation and redistribution dynamics following external exposure, which determine internal exposure profiles.

Mapping internal Hg distributions involves determining both the speciation and concentration of Hg across different tissues and organs. This requires identifying the types, sites, conversion potential, driving forces, and influencing factors of Hg transformations in vivo. The integration of cellular and animal models would be helpful in mapping Hg distributions within the human body. Specifically, the application of stable mercury isotopes in animal models enables accurate quantification of in vivo transformation kinetics and tissue‐specific distributions, and human cell culture systems can provide a controlled environment to pinpoint transformation sites and identify the specific factors influencing these processes. In addition, experimental models should be used to systematically investigate the dose‐time/kinetic relationships of Hg. By combining these experimental results with measured Hg concentrations in human tissues (e.g., blood, urine, hair, and some post‐mortem organs), Hg distributions in humans can be mapped. These experimentally derived data can be integrated with physiologically based pharmacokinetic (PBPK) models, which simulate the absorption, distribution, metabolism, and excretion of chemicals across physiological compartments. The model can describe Hg kinetics, estimate tissue‐specific levels under various exposure scenarios, and identify key organs at risk, thereby improving understanding of internal Hg dynamics in humans.

Currently, studying in vivo Hg transformations presents several challenges. One of these is the simultaneous presence of multiple mediators of Hg transformations, such as enzymes (e.g., catalase and superoxide dismutase) and various ROS (e.g., singlet oxygen and superoxide anion), which complicates the identification of key drivers. Advanced multidisciplinary analytical techniques may provide solutions to these limitations. First, High‐Performance Liquid Chromatography‐Inductively Coupled Plasma‐Mass Spectrometry (HPLC‐ICP‐MS) is more efficient in the identification of potential Hg transformations by distinguishing and quantifying various Hg species simultaneously and can be employed to detect multiple Hg species in animal tissues and cells, thereby distinguishing transformation products and elucidating the chemical pathways involved. In parallel, multi‐omics approaches, such as proteomics and metabolomics, enable the identification of biomolecules that interact with Hg and endogenous factors that affect its transformations, and can also be used to identify potential biomarkers for Hg toxicity, such as oxidative stress‐, DNA damage‐, and inflammation‐related markers. Furthermore, highly sensitive imaging techniques, such as confocal microscopy with Hg‐specific fluorescent probes, synchrotron radiation‐based cryo‐soft X‐ray tomography, and nano‐scale secondary ion mass spectrometry (NanoSIMS), can be applied individually or in combination to enable real‐time and spatially resolved visualization of intracellular Hg and ROS (via ROS‐specific fluorescent labeling), thereby elucidating the role of ROS in Hg transformations and helping to identify the subcellular organelles where such processes occur.

### 4.2 Constructing a Comprehensive Database on Hg Exposure and Tumorigenesis

A comprehensive database on human exposure to environmental pollutants and tumorigenesis is essential for distinguishing the effects of Hg from various confounding factors. This database should include personal information (such as age, gender, and occupation), information on external exposure (including Hg levels and species, as well as other potential tumorigenic factors), and tumor‐related parameters (such as tumor biomarkers and histopathological characteristics). The construction of such a database requires concerted efforts in the following three aspects.

**1) Interdisciplinary collaboration**: A coordinated effort across multiple disciplines is essential, particularly through collaboration among clinical medicine, oncology, public health, environmental health, and environmental toxicology, with each field contributing unique expertise to the investigation of Hg's tumorigenic potential. Clinical medicine provides crucial insights through healthcare records, biomarker data, and biological sample collection. Oncology contributes by analyzing genetic and biomarker data, correlating them with tumor characteristics, and investigating the roles of specific cell types—both tumor‐promoting and tumor‐restraining—in Hg‐associated tumorigenesis, ultimately aiming to identify exposure‐responsive biomarkers and therapeutic entry points. Public health experts can analyze the relationships between exposure and disease at the population level within epidemiological frameworks, using insights from sociology and behavioral science to drive improvements in health behaviors. For example, the Nurses’ Health Study—a long‐term cohort study—has linked environmental and occupational Hg exposures to basal cell carcinoma, squamous cell carcinoma, and melanoma by collecting detailed lifestyle data, conducting regular health assessments, and tracking the exposures of participants over several decades, ultimately linking pollutants to cancer incidence [29]. Environmental health professionals measure pollutant concentrations/speciation (notably Hg) in biological samples, assessing population exposure risks. Finally, environmental toxicologists analyze pollutant effects on cellular/tissue mechanisms, revealing Hg's interactions with the human body and tumorigenic contributions. This integrated approach leverages cross‐disciplinary expertise to build a robust and multifaceted database.

**2) Analytical standardization**: Establishing standardized protocols is equally crucial for the construction of this database. These protocols primarily comprise sampling guidelines for human biological samples and analytical procedures for various Hg species. Sampling protocols should ensure that collection, preservation, and analysis of samples adhere to ethical standards and prevent contamination. Common sources of contamination include unclean containers, airborne Hg or dust, Hg impurities in reagents, cross‐contamination (e.g., blood adhering to tissue surfaces during sampling), and transportation conditions (e.g., exposure to heat, light, or prolonged storage, which may induce Hg transformations). To prevent these issues, standardized contamination‐control protocols should be established, which may also be applied in studies of other contaminants. Furthermore, samples should be preserved under controlled conditions (e.g., long‐term storage of human biological samples for Hg detection at ‐80°C). Regarding Hg analysis, consistent and standardized analytical methods are critical to ensure comparability of Hg measurements across laboratories. Hg in biological tissues is typically analyzed using direct Hg analyzers (DMA‐80), cold vapor atomic fluorescence spectrometry (CVAFS) for THg [57, 58], or gas chromatography‐CVAFS for MeHg [57]. However, inconsistencies in sample pretreatment (e.g., with or without digestion, chemicals used for Hg extraction, and digestion process) and analytical conditions (e.g., instrument stability, detection limits, and performance variability) across labs often result in poor comparability. One study reported interlaboratory variation exceeding 20% for THg and MeHg measurements from identical samples [59]. Notably, no standardized analytical methods currently exist for the speciation of Hg in human tissues, particularly for less commonly monitored species such as mercurous Hg and nanoparticulate Hg. Compared with experimental animal tissues or environmental samples, human tissues present unique analytical challenges. These challenges arise primarily from the limited availability of standard reference materials due to ethical constraints, as well as often extremely low Hg concentrations (e.g., 2.3 µg/kg in brain, 3.3 µg/kg in lung) [60] and complex matrix compositions. For example, blood is widely used as a matrix for biomonitoring, but exhibits high inter‐individual variability in protein‐bound Hg fractions. Studies have shown that Hg can bind to hemoglobin, albumin, and low‐molecular‐weight thiols in both red blood cells and plasma, with species‐dependent distribution patterns [61, 62]. This matrix‐specific binding behavior can significantly influence extraction efficiency and recovery rates during digestion and quantification, resulting in method‐dependent variability. For instance, applying the same acid digestion protocol to fish muscle and cow liver samples spiked with identical Hg concentrations yielded substantially different Hg recovery rates: 100.8% for fish muscle vs. 85.1% for cow liver, showing an approximately 15% difference [63]. Therefore, standardized methods are urgently needed to analyze various Hg species in human biological samples.

**3) Database integration and dynamic updates**: In addition to Hg‐related data (including multi‐omics data reflecting changes in potential biomarkers induced by Hg exposure, as mentioned in Section 4.1), tumor‐related parameters—such as tumor type and biological markers—should also be integrated. By linking both external and internal Hg exposure, along with other tumorigenic factors, to tumor occurrence, this database would serve as a comprehensive platform for assessing Hg‐related tumor risk. Incorporating data on Hg exposure profiles (e.g., Hg distributions and kinetics) and multi‐omics would significantly enhance mechanistic studies, facilitate target identification, and support the development of precise therapeutic interventions. Importantly, the database should be designed as a dynamic and evolving resource, allowing for the incorporation of new environmental pollutants and tumor endpoints as scientific understanding progresses. This iterative updating process will enhance both the accuracy and relevance of identifying tumorigenic factors and improve environmental risk assessment. Moreover, the database should follow standardized protocols and metadata frameworks to facilitate integration into international toxicological platforms—such as Toxicology in the 21st Century (Tox21) or the CompTox Chemicals Dashboard of the U.S. Environmental Protection Agency (EPA)—to support more systematic and interoperable toxicological research. Equally critical is openness and accessibility, which ensures that data can be shared and leveraged across multiple research disciplines. A successful example is the UK Biobank, an open‐access, population‐based cohort platform integrating data from over half a million participants, including imaging data, biomarker data, genetic data, healthcare records, questionnaire data, physical measurements, demographic and lifestyle data, and environmental exposure data. It has also established one of the largest sets of whole‐genome sequences and the world's largest set of protein biomarkers, among othesr resources, collectively supporting over 18,000 peer‐reviewed publications [64]. Drawing on such models, the proposed database should be developed as an open and integrative research infrastructure.

Researchers could leverage this database and employ artificial intelligence (AI)‐based approaches to quantify the contributions of Hg to tumorigenesis while adjusting for confounding factors, including Hg's toxicity modulators (e.g., selenium) and other tumorigenic factors, thereby disentangling Hg‐specific tumorigenic effects in multi‐exposure scenarios. Prior studies have shown that machine learning algorithms within AI frameworks can establish robust aging biomarkers and investigate their associations with cancer susceptibility. This is exemplified by UK Biobank investigations into the Mediterranean lifestyle, physical activity, composite healthy lifestyle scores, and other factors linked to cancer risk [65]. Future integration of demographic data, Hg exposure profiles, and tumor‐related parameters into big data platforms will enable advanced AI applications. For example, similar multi‐source data integration has been used to train AI models in clinical oncology: data from 24 950 patients, including natural language processing annotations, medication, patient‐reported demographic, tumor registry, and genomic data, were integrated at Memorial Sloan Kettering Cancer Center to create a harmonized clinicogenomic dataset. This dataset subsequently trained machine learning models for predicting overall survival [66]. Similarly, by integrating data on Hg speciation and distribution in humans with structured demographic, clinical, and tumor‐related information, AI‐based analyses would enable the prediction of associations between Hg exposure and tumorigenesis.

## 5 Conclusions and Implications

In this study, to advance our understanding of Hg's role in tumorigenesis, we propose an integrative research paradigm that combines in vivo Hg speciation and distribution profiling with data‐driven linkage analysis between Hg exposure and the resulting tumor risk. This dual‐track paradigm enables a more precise understanding of Hg‐induced tumorigenesis and provides the scientific foundation for developing targeted prevention strategies to reduce pollution‐related disease burdens.

While the proposed paradigm is focused on Hg, its core concept—combining in vivo exposure measurements with multi‐dimensional data integration—can be adapted to study other environmental pollutants, including emerging contaminants such as endocrine‐disrupting chemicals (e.g., 1,4‐dioxane and phthalates) and microplastics. Some of these pollutants face challenges similar to those of Hg, including complex exposure patterns [67], limited mechanistic understanding, and difficulties in establishing causal links with disease outcomes [68]. Applying this dual‐track paradigm to these pollutants may help disentangle their specific contributions to tumorigenesis amid complex exposure scenarios, although pollutant‐specific adjustments would be required. Importantly, the paradigm should be viewed as a conceptual framework that integrates experimental evidence and data‐driven analysis to enhance causal inference, rather than a universal solution capable of addressing all challenges. In this context, its applicability depends on chemical properties, biological behaviors, and exposure pathways.

This paradigm would enhance cancer prevention by improving the identification of environmental carcinogens and elucidating their mechanistic pathways. By informing risk assessment and guiding preventive actions, it can also contribute to the achievement of SDG 3 (Good Health and Well‐being), which aims to reduce the global burden of preventable diseases, including cancer. Currently, approximately 44% of global cancer deaths are attributable to estimated risk factors, whereas the remaining 56% cannot be attributed, partly due to the limited identification of environmental carcinogens [69]. Accurate identification of these carcinogens is therefore essential for effective cancer prevention strategies, particularly in low‐ and middle‐income countries (LMICs), where exposure levels are often high and regulatory infrastructure is limited. For example, in many LMICs, artisanal and small‐scale gold mining (ASGM) is widespread and leads to high Hg exposure among local communities. This proposed paradigm addresses key uncertainties in environmental cancer risk assessment by unraveling the carcinogenic potency and mechanistic pathways of pollutants like Hg. This can improve the accuracy of risk estimates, strengthen causal inference, and ultimately support primary prevention. Specifically, this paradigm could facilitate preventive action through 1) exposure surveillance programs to identify and monitor populations with high internal pollutant burdens; 2) public health interventions, including early warning systems and timely interventions for high‐risk groups; 3) international policy coordination by providing scientific evidence for global frameworks, such as the Asturias Declaration, which emphasize environmental cancer prevention.

## Author Contributions

W.T. and H.Z. proposed the topic and structured the framework of the manuscript. S.L. conducted the literature review, wrote the initial draft, and prepared the figures. J.W. and J.C. provided expertise in oncology and contributed to content refinement. R.Z., Z.Y., and T.L. offered writing suggestions and reference materials for the epidemiological sections. H.F. provided constructive input on the environmental toxicology content and revised the figures. N.Z. and L.Q. contributed revision suggestions from a clinical and medical perspective. All authors reviewed and edited the manuscript and approved the final version.

## Conflicts of Interest

Huan Zhong is a roster expert of the MC's Open‐ended Scientific Group.

## Supporting information




**Supporting file**: advs74056‐sup‐0001‐SuppMat.docx

## Data Availability

The authors have nothing to report.
