# Supplementary material for: Paving the Way to Elucidate Hg's Role in Tumorigenesis
Source: Adv Sci (Weinh). 2026 Mar 9;13(30):e14828. doi: 10.1002/advs.202514828 (PMC13248787; doi:10.1002/advs.202514828)
Supplement: Supplementary file 1 — Supporting file: advs74056‐sup‐0001‐SuppMat.docx [file ADVS-13-e14828-s001.docx]

**Supplementary Table 1.** **Epidemiological evidence linking Hg exposure to tumor occurrence.**

| **Organ/body systems** | **Tumor**  **types** | **Study design** | **Population** | **Year** | **Number (M/F)^a^** | **Samples** | **Hg levels**  **(μg/L or μg/kg)^b^** | **Associations^f^** | **Effect Size^g^** | **References** |
| --- | --- | --- | --- | --- | --- | --- | --- | --- | --- | --- |
| Skin | Nonmelanoma | Cross-sectional | National Health and Nutrition Examination Survey (NHANES, the United States) | 2003–2016 | M = 14,237; F = 15,176 | Blood | THg = 1.6 ± 2.4  IHg = 0.3 ± 0.4  MeHg = 1.3 ± 2.4 | Positive | THg Q4 versus Q1: OR 1.79, 95% CI 1.19–2.71, *p* = 0.004; MeHg Q4 versus Q1: OR 1.74, 95% CI 1.13–2.70, *p* = 0.01; IHg: *p* = 0.08 | ^[1]^ |
|  | Basal cell carcinoma (BCC), squamous cell carcinoma (SCC), and melanoma | Cohort | Nurses’ Health Study (NHS) and Health Professionals Follow-up (HPFS) (the United States) | NHS: 1984–2012  HPFS: 1986–2012 | M=6,708; F= 3,730 | Toenail | M: 540 ± 1,410  F: 310 ± 740 | Positive | BCC top versus bottom quintiles: HR 1.34, 95% CI 1.18–1.52, *p* < 0.0001; SCC top versus bottom quartiles: HR 1.41, 95% CI 1.03–1.94, *p* = 0.04; Melanoma top versus bottom quartiles: HR 1.88, 95% CI 1.12–3.16, *p* = 0.02 | ^[2]^ |
| Nervous system | Glioma and meningioma | Cohort | Employed Swedish citizens (Sweden) | 1971–1989 | M = 1,779,646; F = 1,066,346 | Occupation | N.A.^c^ | Positive | No exposure: RR 1.00; Probable exposure: RR 1.76, 95% CI 0.99–3.14 | ^[3]^ |
|  | Glioma | Case-control | Patients with glioma and healthy individuals (Southeastern United States) | 2004–2012 | Cases: M = 182; F = 118  Controls: M = 182; F = 118 | Toenail | Cases: 66 (32–161)  Controls: 69 (31–150) | Non-significant | OR 1.02, 95% CI 0.91–1.14, *p* = 0.70 | ^[4]^ |
| Endocrine system | Thyroid cancer | Cohort | Residents living near industrial complexes (South Korea) | 2003–2011 | M = 1,990; F = 3,223 | Urine | Cases: 1.8  Noncases: 1.2 | Positive | HR = 1.97 (1.03–3.80), *p* = 0.043 (highest versus lowest tertile) | ^[5]^ |
|  | Thyroid tumor | Case-control | Patients with papillary thyroid microcarcinoma, papillary thyroid carcinoma, nodular goiter, and non-diseased individuals (Shenzhen, Guangzhou, China) | 2017–2019 | Cases: M = 72; F = 125  Controls: M = 72; F = 125 | Urine | Cases: 1.01 ± 7.32  Controls: 0.8 ± 3.48 | Non-significant | OR (Q4 versus Q1) = 0.76 (0.39–1.48), *p* = 0.958 | ^[6]^ |
|  | Thyroid cancer | Cross-sectional and Ecological | NHANES (the United States) | 2007–2012 | M = 4,098; F = 3,590 | Soil | 0–32,000 | Positive | N.A. | ^[7]^ |
|  | Pancreatic cancer | Case-control | People with and without pancreatic cancer (Sydney, New South Wales, Australia) | N.A. | Cases: M = 30; F = 15  Controls: M = 17; F = 21 | Pancreatic tissues | N.A. | Positive | *p* = 0.024 (males); *p* = 0.006 (females) | ^[8]^ |
| Respiratory system | Lung cancer | Cohort | Workers exposed to Hg (Madrid, Spain) | 1985–1994 | M = 3,998; F = 0 | Mortality | N.A. | Non-significant | Standardized mortality ratio = 1.03 (95% CI 0.78–1.33) | ^[9]^ |
|  | Lung cancer | Ecological | National Cancer Registry Program (Taiwan, China) | 2001–2005 | N.A. | Soil | 150 | Positive | Soil Hg > 0.23 mg/kg  Males: OR (lung squamous cell carcinoma) = 1.18 (1.08–1.29), *p* < 0.01; OR (lung adenocarcinoma) = 1.04 (0.97–1.13), *p* = 0.28; Females: OR (lung squamous cell carcinoma) = 1.00 (0.80–1.24), *p* = 0.98; OR (lung adenocarcinoma) = 0.91 (0.83–0.99), *p* = 0.02. | ^[10]^ |
|  | Lung cancer | Cohort | Patients with lung cancer (West Pomeranian region of Poland) | 2011–2019 | M = 222; F = 114 | Blood | 1.02 (0.01–6.09) | Positive | Q1 1.55 (1.03–2.34)  Q2 1.20 (0.80–1.79)  Q3 1.49 (0.99–2.22)  Q4 1 (reference) | ^[11]^ |
| Digestive system | Colorectal cancer | Case-control | Research project at the National Cancer  Center (NCC, Republic of Korea) | 2007–2014 | Cases: M = 625; F = 298  Controls: M = 1,250; F = 596 | Dietary Hg intake | Cases: 15.0 ± 2.0  Controls: 14.1 ± 2.2 ^d^ | Positive | Male, low fish and shellfish intake: OR (colorectal cancer) = 3.13 (95% CI 2.33–4.71); high fish and shellfish intake: OR = 3.84 (95% CI 2.20–7.30). Female, low fish and shellfish intake: OR (colorectal cancer) = 2.24 (95% CI 1.36–3.72); OR (colon cancer) = 2.25 (95% CI 1.22–4.16); OR (rectal cancer) = 2.28 (95% CI 1.13–4.57) | ^[12]^ |
|  | Colorectal cancer | Case-control | Patients with colorectal cancer and healthy individuals (Faisalabad, Punjab Province, Pakistan) | 2018–2019 | Cases: M = 86; F = 79  Controls: M = 70; F = 81 | Serum | Cases: 5,501 ± 2,813  Controls: 6,367 ± 2,412 | Non-significant | *p* = Non-significant | ^[13]^ |
|  | Colorectal cancer | Case-control | Patients with colorectal cancer and healthy individuals (Faisalabad, Punjab Province, Pakistan) | 2020–2021 | Cases: M = 88; F = 59  Controls: M = 67; F = 80 | Colorectal tissues | Tumor tissues: 43 (1–715)  Non-tumor tissues: 61 (4–972) | Non-significant | *p* = Non-significant | ^[14]^ |
|  | Colorectal cancer | Case-control | Patients with colorectal cancer (Zvezdara, Belgrade, Serbia) | N.A. | M = 29; F = 30 | Colorectal tissues | Tumor tissues: 0.78 ± 0.7  Adjacent healthy tissues: 2.73 ± 4.35 | Negative | *p* < 0.0001 | ^[15]^ |
|  | Colon cancer | Cohort | Workers exposed to Hg (Madrid, Spain) | 1985–1994 | M = 3,998; F = 0 | Mortality | N.A. | Negative | N.A. | ^[9]^ |
|  | Liver cancer | Cohort | Workers exposed to Hg (Madrid, Spain) | 1985–1994 | M = 3,998; F = 0 | Mortality | N.A. | Positive | Standardized mortality ratio = 1.25 (95% CI 0.72–2.03) | ^[9]^ |
|  | Gallbladder cancer | Case-control | Patients with gallbladder pre-malignant and malignant conditions (PM-M), and chronic cholecystitis (C-C) (Bankura, West Bengal, India) | 2012–2016 | PM-M: M = 11; F = 35  C-C: M = 15; F = 50 | Gallstones | PM-M: 58,760 ± 11,040  C-C: 45,770 ± 21,210 | Positive | *p* < 0.001 | ^[16]^ |
| Urinary system | Prostate cancer | Case-control | Patients with benign prostatic hyperplasia (BPH), prostate cancer (PCa), and healthy individuals (Taiwan, China) | N.A. | Patients with BPH: M = 17; F = 0  Patients with PCa: M = 20; F = 0  Controls: M = 23; F = 0 | Blood | Patients with BPH: 1.84 ± 1.75  Patients with PCa: 6.9 ± 9  Controls: 1.79 ± 1.7 | Positive | PCa: OR 101.7 (95% CI 1.21–8538), *p* = 0.041; BPH: OR 17.75 (95% CI 1.05–301.4), *p* = 0.007 | ^[17]^ |
|  | Prostate cancer | Case-control | Patients with benign prostatic hyperplasia (BPH), prostate cancer (PCa), and healthy controls (HC) (Faisalabad, Punjab Province, Pakistan) | 2020–2021 | Patients with BPH: M = 188; F = 0  Patients with PCa: M = 217; F = 0  Controls: M = 233; F = 0 | Serum | Patients with BPH: 29.65 ± 0.02  Patients with PCa: 17.31 ± 0.02  Controls: 39.45 ± 0.02 | Negative | BPH versus HC: *p* = 0.041; PC versus HC: *p* = 0.01; BPH versus PC: *p* = 0.000 | ^[18]^ |
|  | Prostate cancer | Case-control | Croatian cohort and Serbian cohort (Croatia and Serbia) | 2011–2013 | Cases: M = 103; F = 0  Controls: M = 91; F = 0 | Blood | Cases: 7.1  Controls: 2.92 | Positive | *p* < 0.0001 | ^[19]^ |
|  | Renal cell carcinoma | Case-control | Patients with renal cell carcinoma and control individuals (India) | 2019–2021 | Cases: M = 62; F = 14  Controls: M = 51; F = 13 | Blood | Cases: 3.67 ± 3.54  Controls: 0.36 ± 0.91 | Positive | *p* = 0.001 | ^[20]^ |
|  | Renal cell carcinoma | Case-control | Patients with renal cell carcinoma and control individuals (India) | 2019–2021 | Cases: M = 62; F = 14  Controls: M = 51; F = 13 | Urine | Cases: 3.83 ± 18.16  Controls: 0.28 ± 0.56 | Non-significant | *p* = 0.12 | ^[20]^ |
|  | Kidney cancer | Cohort | Workers exposed to Hg (Madrid, Spain) | 1985–1994 | M = 3,998; F = 0 | Mortality | N.A. | Negative | N.A. | ^[9]^ |
|  | Bladder cancer | Case-control | Patients with bladder cancer (BCa) and healthy controls | N.A. | Discovery cohort: patients with BCa (M = 2; F = 4),  controls (M+F=12);  Validation cohort: patients with BCa (M = 5; F = 16),  non-tumor bladder patients (M+F=29), controls (M+F=18) | Serum | N.A. | Positive | N.A. | ^[21]^ |
|  | Bladder cancer | Cohort | Workers exposed to Hg (Madrid, Spain) | 1985–1994 | M = 3,998; F = 0 | Mortality | N.A. | Negative | N.A. | ^[9]^ |
| Reproductive system | Breast cancer | Cohort | National Institute of Environmental Health Sciences (NIEHS) sister Study (the United States) | 2003–2009 | M = 0; F = 50,884 | Air | N.A. | Positive | Q5 versus Q1, HR = 1.2, 95% CI: 1.0–1.4; *p* = 0.03 | ^[22]^ |
|  | Breast cancer | Cross-sectional | NHANES (the United States) | 2003–2012 | M = 0; F = 9,260 | Blood | Cases: 1.02  Controls: 0.94 | Non-significant | Q4 versus Q1: OR = 1.27 (95% CI 0.82–1.97) | ^[23]^ |
|  | Breast cancer | Case-only | Patients with cancer (Chicagoland area, the United States) | 2005–2008 | M = 0; F = 989 | Air | N.A. | Non-significant | Q5 versus Q1: OR = 1.0 (95% CI 0.5–2.1), *p* = 0.23. | ^[24]^ |
|  | Breast cancer | Ecological | Surveillance, Epidemiology, and End Results (SEER) Program (the United States) | 1973–2014 | Covering approximately 34.6% of the U.S. population | Air | Hg emission density: 0.84^e^ | Positive | β = 19.84 (95% CI 6.96–32.72), *p* = 0.003 | ^[25]^ |

^a^ M and F represent male and female, respectively.

^b^ Hg concentrations are expressed as μg/L in blood, urine, and serum; μg/kg dry weight in toenail, hair, and soil; and μg/kg wet weight in tumor and healthy tissues.

^c^ Data are not available.

^d^ Hg intake levels are expressed in μg/day.

^e^ Hg emission density is expressed in lb/square mile.

^f^ “Positive”, “non-significant”, and “negative” refer to statistically significant positive, non-significant, and statistically significant negative correlations, respectively, between tumor incidence and Hg concentrations (in environmental or biological samples).

^g^ Q1–Q5: Quartiles 1–5, OR: Odds Ratio, CI: Confidence Interval, *p*: *p*-value (probability value), HR: Hazard Ratio, RR: Relative Risk, β: Beta coefficient.

**Supplementary Table 2. Potential pathways underlying Hg-induced tumorigenesis.**

| **Potential pathways** | **Hg species^a^** | **Exposure concentration (μg/L or μg/kg)** | **Exposure duration (days)** | **Exposure**  **routes** | **Cellular models** | **Animal models^e^** | **Key molecular alterations** | **References** |
| --- | --- | --- | --- | --- | --- | --- | --- | --- |
| Oxidative stress and inflammation | Hg(II) and MeHg | 100, 500, and 1,000 | 11 | *In vitro* exposure | Human intestinal epithelial cells (Caco-2 and HT29-MTX) |  | Increased IL-8 (15–126%) and IL-1β (39–63%) levels through M1 macrophage polarization; elevated ROS/RNS levels (44–140%); upregulated expression of stress proteins; disruption of tight junctions; enhanced paracellular permeability (123–170%); activation of p38 MAPK, JNK, and NF-κB signaling pathways | ^[26]^ |
|  | Hg(II) | 2.01 | 1 | *In vitro* exposure | Human keratinocytes |  | Decreased GJIC; reduced formation and secretion of IL-1β and TNFα | ^[27]^ |
|  | Hg(II) and MeHg | 200.6 and 1003 | 1 | *In vitro* exposure | *Caenorhabditis elegans* |  | Induced hydrogen peroxide (H_2_O_2_), which cause bulky DNA lesions and oxidative DNA damage | ^[28]^ |
|  | Hg(II) | 401.2–4,012 | 1 | *In vitro* exposure | HIT-T15 cells |  | Oxidative stress-induced mitochondrial membrane depolarization, cytochrome c release, PARP and caspase-3 activation, ATP depletion, increased LDH release, and impaired insulin secretion | ^[29]^ |
|  | MeHg | 200.6–4,012 | 1 | *In vitro* exposure | HIT-T15 cells | Mouse pancreatic islets | ROS-mediated mitochondrial dysfunction characterized by membrane depolarization, cytochrome c release, and caspase-3 activation; suppressed insulin secretion | ^[30]^ |
|  | Hg(II) | 300 | 7 | Waterborne exposure |  | *Channa punctatus* (intestine) | Decreased phagocytosis, nitric oxide (NO) and myeloperoxidase (MPO) production; increased pro-inflammatory cytokines TNF-α and IL-6; mitochondrial degeneration | ^[31]^ |
|  | Hg(II) and MeHg | 1,000, 5,000, and 10,000 | 122 | Oral via drinking water |  | Balb/c mice (small intestine and colon tissues) | Oxidative stress in small intestine and colon; inflammation mainly in colon; increased fecal albumin; increased *Muc2* expression; p38 MAPK activation and increased crypt depth (MeHg only); decreased microbial-derived short-chain fatty acids | ^[32]^ |
|  | Hg(II) | 45 | 5, 10, and 15 | Waterborne exposure |  | *Perna viridis* (digestive gland) | Increased lipid peroxidation, protein carbonyls, hydrogen peroxide; DNA damage; modulated antioxidant enzymes (SOD, CAT, GPx, GR, GST); elevated GSH, ascorbic acid, and metallothionei | ^[33]^ |
|  | Hg(II) | 5.01, 20.1, and 5,015 | 1, 3, and 7 | *In vitro* exposure | Rat cortical neurons |  | Neurite degeneration and network disruption; increased inward synaptic currents and membrane depolarization; 2–5-fold rise in intracellular calcium concentration; disruption of β-tubulin | ^[34]^ |
|  | MeHg | 0.30, 1.5, and 3 | 1 and 2 | Cortical injection |  | Wistar rats (frontal cortex) | Increased DNA damage | ^[35]^ |
|  | Hg(II) | Initial 4.60, then 0.49 per week^b^ | 30 and 60 | Intramuscular injection |  | Wistar rats (kidney) | Renal oxidative stress | ^[36]^ |
|  | Hg(0) | 4,000^c^ | 10 | Inhalation exposure |  | Long-Evans rats (lung) | Increased expression of inflammatory genes (TNFα, TNFR1, IL-2, IL-7, prostaglandin E2 receptor), heat shock proteins, GST-pi, mGST1, metallothionein, thioredoxin peroxidase, MRP, P-gp, ZnT1, PKC, and epidermal FABP; decreased expression of c-jun/AP-1 and PI3K | ^[37]^ |
|  | MeHg | 500, 1,000, and 3,000 per day^d^ | 14 | Oral gavage |  | Wistar rats (prostate) | Chronic inflammation, stratified epithelial hyperplasia, epithelial inflammatory reactive atypia and epithelial atrophy | ^[38]^ |
|  | Hg(II) | Initial dose 4.6, then 0.07 per day^b^ | 30 | Injection |  | Wistar rats (prostate) | Increased lipid peroxidation; altered antioxidant enzyme activities (SOD, CAT) and nonprotein thiol levels | ^[39]^ |
|  | Hg(II) | 2,000 and 4,000 per day^d^ | 60 | Oral gavage |  | Wistar rats (thyroid) | Increased lipid peroxidation; decreased antioxidative enzymes (SOD, GPx, GR, CAT, GSH) and metabolic enzymes (SDH, ATPase, ACPase, ALPase) | ^[40]^ |
|  | Hg(II) | 40, 80, 160 | 4 | Waterborne exposure |  | *Holothuria forskali* (intestine) | Increased MDA, protein carbonyls (PCO), advanced oxidation protein products (AOPP); increased glutathione (GSH), vitamin C, non-protein thiols (NPSH); increased antioxidant enzyme activities (CAT, SOD, GPx); increased metallothionein (MT); DNA degradation (genotoxicity); decreased AChE activity | ^[41]^ |
| Abnormal proliferation and enhanced survival | Hg(II) | 20.1 | 1 | *In vitro* exposure | HaCaT cells and PLB-985 cells |  | Increased cell proliferation mediated by elevated NADPH oxidase activity | ^[42]^ |
|  | MeHg | 100.3–200.6 | 14 | *In vitro* exposure | MCF-7 breast cancer cells |  | Increased intracellular Ca^2+^ levels and enhanced Erk1/2 phosphorylation | ^[43]^ |
|  | Hg(II) | 200.6 | 1 | *In vitro* exposure | MCF-7 breast cancer cells |  | Promoted tumor cell proliferation through activation of estrogen receptor-α (ERα) signaling | ^[44]^ |
|  | MeHg | 20.1 | 18 | *In vitro* exposure | Non-tumorigenic thyroid cells (Nthy-ori-3-1) |  | Promoted thyroid cell proliferation by increasing ERK phosphorylation and inducing G2/M phase accumulation. | ^[45]^ |
|  | MeHg | 401.2 | 0.25 | *In vitro* exposure | SH-SY5Y cells |  | Upregulation of Bcl-2 via Akt/CREB activation due to PTEN S-mercuration | ^[46]^ |
| Epigenetic  alterations | MeHg | 0.2 | 6 | *In vitro* exposure | LUHMES Cells |  | Increased DNA methylation and DNA methyltransferase 1 (DNMT1) levels | ^[47]^ |
|  | MeHg | 0.5 and 1 | 2 | *In vitro* exposure | Rat embryonic cortical NSCs |  | Decreased global DNA methylation levels | ^[48]^ |
|  | MeHg | 3,000^b^ per day | 5–7 | Oral administration |  | Pregnant C57BL/6J mice (fetal brain tissue) | Increased DNA methylation and DNA methyltransferase 1 (DNMT1) levels | ^[47]^ |
|  | MeHg | 500 and 5,000 | 1 | Oral gavage |  | Wistar rats  (kidney) | Increased demethylation of critical CpGs in the first exon of MMP9, decreased binding of MeCP2 and PEA3, and increased MMP9 expression were observed | ^[49]^ |
|  | MeHg | 2 | 0.17–1 | Waterborne exposure |  | Zebrafish | Altered DNA methylation | ^[50]^ |

^a^ Hg(II): divalent inorganic mercury; MeHg: methylmercury; MeHgOH: methylmercury hydroxide; Hg(0): elemental mercury.

^b^ Hg concentration is expressed in μg/kg.

^c^ Hg concentration is expressed in μg/m^3^.

^d^ Hg concentration is expressed in μg/kg body weight.

^e^ Organs or tissues in parentheses indicate the primary targets analyzed in animal models.

**Supplementary References**

1 J. Rhee, T. M. Vance, R. Lim, D. C. Christiani, A. A. Qureshi, E. Cho. Association of blood mercury levels with nonmelanoma skin cancer in the USA using National Health and Nutrition Examination Survey data (2003-2016). *Brit. J. Dermatol.* 2020, **183**, 480.

2 N. H. Matthews, M. Koh, W. Q. Li, T. Li, W. C. Willett, M. J. Stampfer, D. C. Christiani, J. S. Morris, A. A. Qureshi, E. Cho. A Prospective Study of Toenail Trace Element Levels and Risk of Skin Cancer. *Cancer Epidemiol. Biomarkers Prev*. 2019, **28**, 1534.

3 A. Navas-Acién, M. Pollán, P. Gustavsson, N. Plato. Occupation, exposure to chemicals and risk of gliomas and meningiomas in Sweden. *Am. J. Ind. Med.* 2002, **42**, 214.

4 J. H. Creed, N. C. Peeri, G. M. Anic, R. C. Thompson, J. J. Olson, R. LaRocca, S. A. Chowdhary, J. D. Brockman, T. A. Gerke, L. B. Nabors, K. M. Egan. Methylmercury exposure, genetic variation in metabolic enzymes, and the risk of glioma. *Sci. Rep*. 2019, **9**, 10861.

5 S. Kim, S. H. Song, C. W. Lee, J. T. Kwon, E. Y. Park, J. K. Oh, H. J. Kim, E. Park, B. Kim. Low-Level Environmental Mercury Exposure and Thyroid Cancer Risk Among Residents Living Near National Industrial Complexes in South Korea: A Population-Based Cohort Study. *Thyroid* 2022, **32**, 1118.

6 M. Liu, J. Y. Song, Y. S. Jiang, Y. Liu, J. L. Peng, H. W. Liang, C. Wang, J. Jiang, X. J. Liu, W. Wei, J. Peng, S. Liu, Y. M. Li, N. Xu, D. X. Zhou, Q. H. Zhang, J. Q. Zhang. A case-control study on the association of mineral elements exposure and thyroid tumor and goiter. *Ecotox. Environ. Safe*. 2021, **208**, 111615.

7 Y. Shaked, J. Yang, M. Monaghan, M. van Gerwen. The Association between Metals and Thyroid Cancer in Puerto Rico-A National Health and Nutrition Examination Survey Analysis and Ecological Study. *Toxics* 2024, **12**, 632.

8 R. Pamphlett, A. J. Colebatch, P. A. Doble, D. P. Bishop. Mercury in Pancreatic Cells of People with and without Pancreatic Cancer. *Int. J. Environ. Res. Public Health* 2020, **17**, 8990.

9 M. G. Gómez, P. Boffetta, J. D. C. Klink, S. Español, J. G. Quintana, D. Colin. Cancer mortality in mercury miners. *Gac. Sanit*. 2007, **21**, 210.

10 H. H. Huang, J. Y. Huang, C. C. Lung, C. L. Wu, C. C. Ho, Y. H. Sun, P. C. Ko, S. Y. Su, S. C. Chen, Y. P. Liaw. Cell-type specificity of lung cancer associated with low-dose soil heavy metal contamination in Taiwan: An ecological study. *BMC Public Health* 2013, **13**, 330.

11 S. Pietrzak, J. Wójcik, P. Baszuk, W. Marciniak, M. Wojtys, T. Debniak, C. Cybulski, J. Gronwald, J. Alchimowicz, B. Masojc, P. Waloszczyk, D. Gajic, T. Grodzki, A. Jakubowska, R. J. Scott, J. Lubinski, M. R. Lener. Influence of the Levels of Arsenic, Cadmium, Mercury and Lead on Overall Survival in Lung Cancer. *Biomolecules* 2021, **11**, 1160.

12 H. Kim, J. Lee, H. D. Woo, D. W. Kim, J. H. Oh, H. J. Chang, D. K. Sohn, A. Shin, J. Kim. Dietary mercury intake and colorectal cancer risk: A case-control study. *Clin. Nutr*. 2020, **39**, 2106.

13 M. H. R. Mahmood, M. A. Qayyum, F. Yaseen, T. Farooq, Z. Farooq, M. Yaseen, A. Irfan, K. Muddassir, M. N. Zafar, M. T. Qamar, A. M. Abbasi, H. Y. Liu. Multivariate Investigation of Toxic and Essential Metals in the Serum from Various Types and Stages of Colorectal Cancer Patients. *Biol. Trace Elem. Res*. 2022, **200**, 31.

14 M. A. Qayyum, T. Farooq, A. Baig, T. H. Bokhari, M. N. Anjum, M. H. U. R. Mahmood, A. R. Ashraf, K. Muddassir, M. Ahmad. Assessment of essential and toxic elemental concentrations in tumor and non-tumor tissues with risk of colorectal carcinoma in Pakistan. *J. Trace Elem. Med. Biol. 2023*, **79**, 127234.

15 J. T. Juloski, A. Rakic, V. V. Cuk, V. M. Cuk, S. Stefanovic, D. Nikolic, S. Jankovic, A. M. Trbovich, S. R. De Luka. Colorectal cancer and trace elements alteration. *J. Trace Elem. Med. Biol*. 2020, **59**, 126451.

16 B. Mondal, D. Maulik, M. Mandal, G. N. Sarkar, S. Sengupta, D. Ghosh. Analysis of Carcinogenic Heavy Metals in Gallstones and its Role in Gallbladder Carcinogenesis. *J. Gastrointest. Canc*. 2017, **48**, 361.

17 W. H. Chang, C. C. Lee, Y. H. Yen, H. L. Chen. Oxidative damage in patients with benign prostatic hyperplasia and prostate cancer co-exposed to phthalates and to trace elements. *Environ. Int*. 2018, **121**, 1179.

18 M. A. Qayyum, M. H. R. Mahmood, T. Farooq, A. Irfan, S. Iqbal, N. Hussain. Multivariate Statistical Evaluation of 20 Metals/Metalloid Levels in the Serum of Patients with Prostate Gland Diseases. *Indian J. Clin. Biochem*. 2024, **40**, 392.

19 A. Pizent, M. Andelkovic, B. T. Lovakovic, T. Z. Semren, A. B. Djordjevic, M. Gamulin, V. Bonderovic, M. Acimovic, Z. Bulat. Environmental Exposure to Metals, Parameters of Oxidative Stress in Blood and Prostate Cancer: Results from Two Cohorts. *Antioxidants* 2022, **11**, 2044.

20 S. Panaiyadiyan, J. A. Quadri, B. Nayak, S. Pandit, P. Singh, A. Seth, A. Shariff. Association of heavy metals and trace elements in renal cell carcinoma: A case-controlled study. *Urol. Oncol.* 2022, **40**, 111.e11-111.e18.

21 S. Wach, K. Weigelt, B. Michalke, V. Lieb, R. Stoehr, B. Keck, A. Hartmann, B. Wullich, H. Taubert, A. Chaudhri. Diagnostic potential of major and trace elements in the serum of bladder cancer patients. *J. Trace Elem. Med. Biol*. 2018, **46**, 150.

22 A. J. White, K. M. O'Brien, N. M. Niehoff, R. Carroll, D. P. Sandler. Metallic Air Pollutants and Breast Cancer Risk in a Nationwide Cohort Study. *Epidemiology* 2019, **30**, 20.

23 Y. D. Wei, J. M. Zhu. Blood levels of endocrine-disrupting metals and prevalent breast cancer among US women. *Med. Oncol*. 2020, **37**, 1.

24 J. K. Kresovich, S. Erdal, H. Y. Chen, P. H. Gann, M. Argos, G. H. Rauscher. Metallic air pollutants and breast cancer heterogeneity. *Environ. Res*. 2019, **177**, 108639.

25 V. Vu, N. Navalkar, Y. D. Wei. Endocrine-disrupting metals in ambient air and female breast cancer incidence in US. *Gynecol. Endocrinol*. 2019, **35**, 1099.

26 P. Rodríguez-Viso, A. Domene, D. Vélez, V. Devesa, V. Monedero, M. Zúñiga. Mercury toxic effects on the intestinal mucosa assayed on a bicameral model: Possible role of inflammatory response and oxidative stress. *Food Chem. Toxicol.* 2022, **166**, 113224.

27 R. Zefferino, A. Leone, S. Piccaluga, R. Cincione, L. Ambrosi. Mercury modulates interplay between IL-1β, TNF-α, and gap junctional intercellular communication in keratinocytes: mitigation by lycopene. *J. Immunotoxicol*. 2008, **5**, 353.

28 L. H. Wyatt, A. L. Luz, X. Cao, L. L. Maurer, A. M. Blawas, A. Aballay, W. K. Y. Pan, J. N. Meyer. Effects of methyl and inorganic mercury exposure on genome homeostasis and mitochondrial function in *Caenorhabditis elegans*. *DNA Repair* 2017, **52**, 31.

29 Y. W. Chen, C. F. Huang, C. Y. Yang, C. C. Yen, K. S. Tsai, S. H. Liu. Inorganic mercury causes pancreatic β-cell death via the oxidative stress-induced apoptotic and necrotic pathways. *Toxicol. Appl. Pharmacol.* 2010, **243**, 323.

30 Y. W. Chen, C. F. Huang, K. S. Tsai, R. Sen Yang, C. C. Yen, C. Y. Yang, S. Y. Lin-Shiau, S. H. Liu. Methylmercury induces pancreatic β-cell apoptosis and dysfunction. *Chem. Res. Toxicol.* 2006, **19**, 1080.

31 M. Begam, M. Sengupta. Immunomodulation of intestinal macrophages by mercury involves oxidative damage and rise of pro-inflammatory cytokine release in the fresh water fish Channa punctatus Bloch. *Fish Shellfish Immunol.,* 2015, **45**, 378.

32 P. Rodríguez-Viso, A. Domene, D. Vélez, V. Devesa, V. Monedero, M. Zúñiga. Oral exposure to inorganic mercury or methylmercury elicits distinct pro-inflammatory and pro-oxidant intestinal responses in a mouse model system. *Food Chem. Toxicol.* 2023, **177**, 113801.

33 X. N. Verlecar, K. B. Jena, G. B. N. Chainy. Modulation of antioxidant defences in digestive gland of *Perna viridis* (L.), on mercury exposures. *Chemosphere* 2008, **71**, 1977.

34 F. Xu, S. Farkas, S. Kortbeek, F.-X. Zhang, L. Chen, G. W. Zamponi, N. I. Syed. Mercury-induced toxicity of rat cortical neurons is mediated through N-methyl-D-Aspartate receptors. *Mol. Brain* 2012, **5**, 30.

35 B. I. Juárez, H. Portillo-Salazar, R. González-Amaro, P. Mandeville, J. R. Aguirre, M. E. Jiménez. Participation of N-methyl-D-aspartate receptors on methylmercury-induced DNA damage in rat frontal cortex. *Toxicology* 2005, **207**, 223.

36 E. C. de Almeida, V. D. Faria, F. D. Cirineu, M. G. A. Santiago, B. Miotto, J. C. S. Vieira, C. P. Braga, J. Adamec, A. A. H. Fernandes, M. A. R. Buzalaf, P. D. Padilha. Metalloproteomic Investigation of Hg-Binding Proteins in Renal Tissue of Rats Exposed to Mercury Chloride. *Int. J. Mol. Sci.* 2024, **25**, 164.

37 J. Liu, D. Lei, M. P. Waalkes, R. P. Beliles, D. L. Morgan. Genomic analysis of the rat lung following elemental mercury vapor exposure. *Toxicol. Sci*. 2003, **74**,174.

38 D. A. F. da Silva, F. Barbosa, W. R. Scarano. Oral exposure to methylmercury modifies the prostatic microenvironment in adult rats. *Int. J. Exp. Pathol.* 2012, **93**, 354.

39 C. S. Martinez, A. G. Escobar, J. G. D. Torres, D. S. Brum, F. W. Santos, M. J. Alonso, M. Salaices, D. V. Vassallo, F. M. Peçanha, F. G. Leivas, G. A. Wiggers. Chronic Exposure to Low Doses of Mercury Impairs Sperm Quality and Induces Oxidative Stress in Rats. *J. Toxicol. Environ. Health A.* 2014, **77**, 143.

40 M. V. Rao, B. Chhunchha. Protective role of melatonin against the mercury induced oxidative stress in the rat thyroid. *Food Chem. Toxicol.* 2010, **48**, 7.

41 I. Rabeh, K. Telahigue, S. Bejaoui, T. Hajji, L. Chouba, M. EL Cafsi, N. Soudani. Effects of mercury graded doses on redox status, metallothionein levels and genotoxicity in the intestine of sea cucumber. *Chem. Ecol.* 2019, **35**, 204.

42 A. Mohammadi-Bardbori, A. Rannug. Arsenic, cadmium, mercury and nickel stimulate cell growth via NADPH oxidase activation. *Chem. Biol. Interact.* 2014, **224**, 183.

43 O. A. Sukocheva, Y. Yang, J. F. Gierthy, R. F. Seegal. Methyl mercury influences growth-related signaling in MCF-7 breast cancer cells. *Environ. Toxicol.* 2005, **20**, 32.

44 M. B. Martin, R. Reiter, T. Pham, Y. R. Avellanet, J. Camara, M. Lahm, E. Pentecost, K. Pratap, B. A. Gilmore, S. Divekar, R. S. Dagata, J. L. Bull, A. Stoica. Estrogen-like activity of metals in Mcf-7 breast cancer cells. *Endocrinology*. 2003, **144**, 2425.

45 V. Maggisano, S. Bulotta, M. Celano, J. Maiuolo, S. M. Lepore, L. Abballe, M. Iannone, D. Russo. Low Doses of Methylmercury Induce the Proliferation of Thyroid Cells In Vitro Through Modulation of ERK Pathway. *Int. J. Mol. Sci.* 2020, **21**, 1556.

46 T. Unoki, Y. Abiko, T. Toyama, T. Uehara, K. Tsuboi, M. Nishida, T. Kaji, Y. Kumagai. Methylmercury, an environmental electrophile capable of activation and disruption of the Akt/CREB/Bcl-2 signal transduction pathway in SH-SY5Y cells. *Sci. Rep.* 2016, **6**, 28944.

47 S. Go, H. Kurita, M. Hatano, K. Matsumoto, H. Nogawa, M. Fujimura, M. Inden, I. Hozumi. DNA methyltransferase- and histone deacetylase-mediated epigenetic alterations induced by low-level methylmercury exposure disrupt neuronal development. *Arch. Toxicol.* 2021, **95**, 1227.

48 R. Bose, N. Onishchenko, K. Edoff, A. M. Janson Lang, S. Ceccatelli. Inherited Effects of Low-Dose Exposure to Methylmercury in Neural Stem Cells. *Toxicol. Sci*. 2012, **130**, 383**.**

49 H. Khan, R. D. Singh, R. Tiwari, S. Gangopadhyay, S. K. Roy, D. Singh, V. Srivastava. Mercury exposure induces cytoskeleton disruption and loss of renal function through epigenetic modulation of MMP9 expression. *Toxicology* 2017, **386**, 28.

50 M. J. Carvan III, in *Behavioral and Neural Genetics of Zebrafish* (Ed.: R. T. Gerlai), Academic Press, 2020, pp. 493–510.
